# Dynamic spatiotemporal modeling of the infected rate of visceral leishmaniasis in human in an endemic area of Amhara regional state, Ethiopia

**DOI:** 10.1371/journal.pone.0212934

**Published:** 2019-03-01

**Authors:** Anteneh Asmare Godana, Samuel Musili Mwalili, George Otieno Orwa

**Affiliations:** 1 Pan African University Institute for Basic Science, Technology and Innovation (PAUSTI), Nairobi, Kenya; 2 Jomo Kenyatta University of Agriculture and Technology, Department of Statistics and Actuarial Sciences, Nairobi, Kenya; University of Kwazulu-Natal, SOUTH AFRICA

## Abstract

Visceral Leishmaniasis is a very dangerous form of leishmaniasis and, shorn of appropriate diagnosis and handling, it leads to death and physical disability. Depicting the spatiotemporal pattern of disease is important for disease regulator and deterrence strategies. Spatiotemporal modeling has distended broad veneration in recent years. Spatial and spatiotemporal disease modeling is extensively used for the analysis of registry data and usually articulated in a hierarchical Bayesian framework. In this study, we have developed the hierarchical spatiotemporal Bayesian modeling of the infected rate of Visceral leishmaniasis in Human (VLH). We applied the Stochastics Partial Differential Equation (SPDE) approach for a spatiotemporal hierarchical model for Visceral leishmaniasis in human (VLH) that involves a GF and a state process is associated with an autoregressive order one temporal dynamics and the spatially correlated error term, along with the effect of land shield, metrological, demographic, socio-demographic and geographical covariates in an endemic area of Amhara regional state, Ethiopia. The model encompasses a Gaussian Field (GF), affected by an error term, and a state process described by a first-order autoregressive dynamic model and spatially correlated innovations. A hierarchical model including spatially and temporally correlated errors was fit to the infected rate of Visceral leishmaniasis in human (VLH) weekly data from January 2015 to December 2017 using the R package R-INLA, which allows for Bayesian modeling using the stochastic partial differential equation (SPDE) approach. We found that the mean weekly temperature had a significant positive association with infected rate of VLH. Moreover, net migration rate, clean water coverage, average number of households, population density per square kilometer, average number of persons per household unit, education coverage, health facility coverage, mortality rate, and sex ratio had a significant association with the infected rate of visceral leishmaniasis (VLH) in the region. In this study, we investigated the dynamic spatiotemporal modeling of Visceral leishmaniasis in Human (VLH) through a stochastic partial differential equation approach (SPDE) using integrated nested Laplace approximation (INLA). Our study had confirmed both metrological, demographic, sociodemographic and geographic covariates had a significant association with the infected rate of visceral leishmaniasis (VLH) in the region.

## Introduction

**Fig 1:**
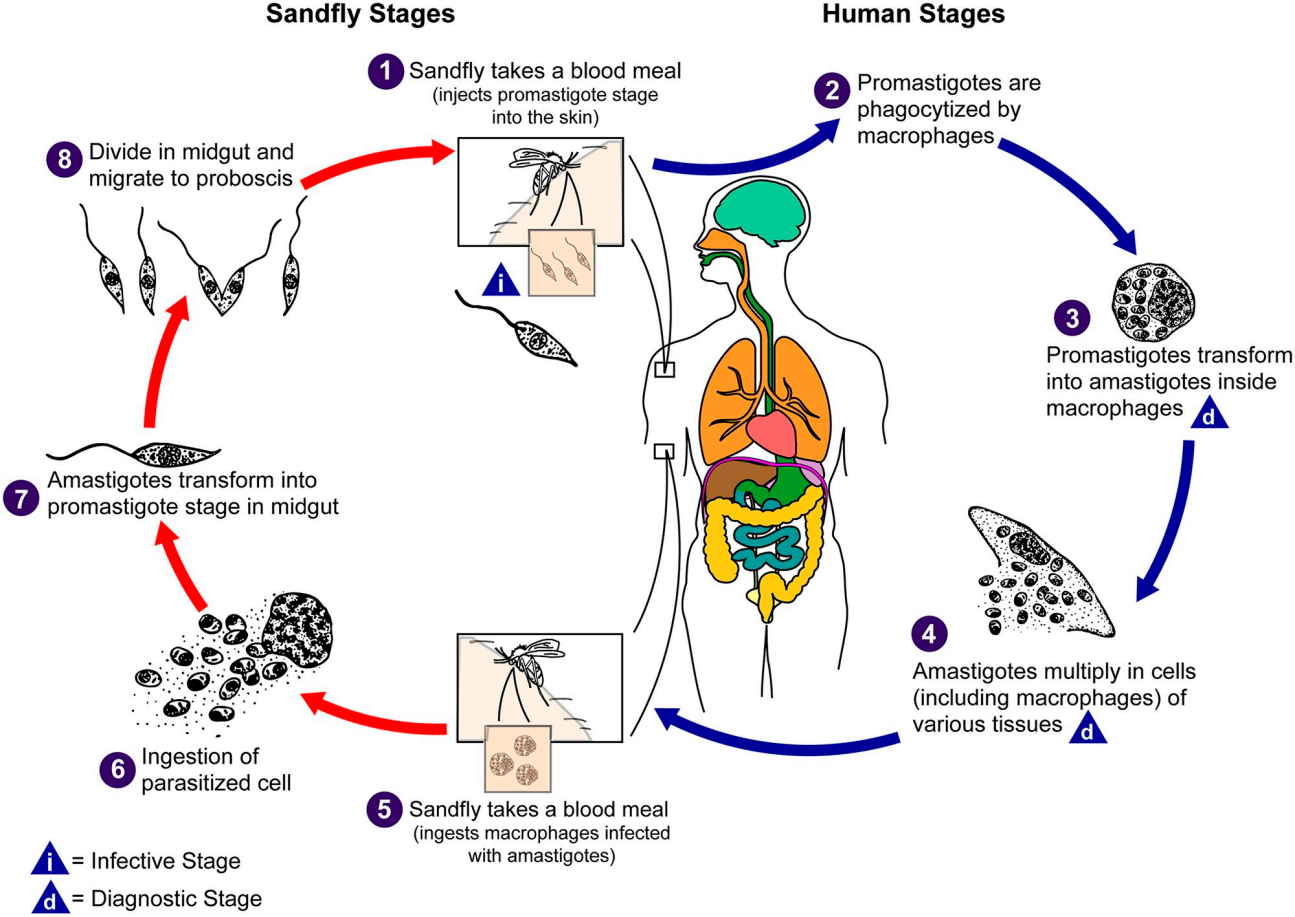
Life cycles of leishmaniasis. (**Source**:https://www.cdc.gov/parasites/leishmaniasis/biology.html, [6].

Visceral leishmaniasis is the second most parasitic killer in the globe next to malaria, responsible for an estimated 200,000 to 400,000 infections each year globally [1, 2]. Globally about 90-95% of visceral leishmaniasis infections is contributed by most Sub-saharan countries, Asian and Latin American, According to the study by [3, 4]. Based on the species of Leishmania parasites and other immunological and epidemiological aspects, Leishmania infection can lead to cutaneous (CL), mucocutaneous (MCL) or visceral leishmaniasis (VL) [5]. Leishmaniasis is a group of diseases caused by protozoan parasites of the genus Leishmania that are transmitted between humans and other mammalian hosts by phlebotomine sand flies [5], this human infection caused by over 20 species [5, 6]. Furthermore, Fig 1 below shows that the life cycles of parasite of Leishmaniasis diseases. Visceral Leishmaniasis is a very dangerous form of leishmaniasis and, shorn of appropriate diagnosis and handling, it leads to death and physical disability [7]. Depicting the spatiotemporal pattern of disease is important for disease regulator and deterrence strategies. Spatiotemporal modeling has distended broad veneration in recent years. Spatial and spatiotemporal disease modeling is extensively used for the analysis of registry data and usually articulated in a hierarchical Bayesian framework. Integrated nested Laplace approximations (INLA) would be used as an apparatus for Bayesian inference. INLA is a favorable substitute to Markov chain Monte Carlo (MCMC) methods which deliver very exact results within little computational period [8], [9]. In the spatiotemporal scheme, data coming from different locations are assumed to be the realization of a contentiously indexed spatial process or random field changing in time; Thus;

$$\begin{array}{c} Y\left(S:t\right):S\in D_{s}\in R^{m},t\in D_{t}\in R \end{array}$$ (1)

*Y*(*S*: *t*) denote a spatiotemporal random process, where D_*s*_ is the spatial domain of interest, D_*t*_ is the temporal domain of interest, *s* is a spatial index and *t* a time index. Gaussian Fields (GFs) is a prominent application in spatial statistics and spatiotemporal modeling [8], [10], [11], and it is a basic form of constructing hierarchical spatiotemporal Bayesian modeling [8]. Let *S* ∈ *D* ⊂ R^*m*^ is a continuous indexed Gaussian Field (GF) for all finite collection of [*X*(*s*_*i*_)] are jointly Gaussian distribution. The Gaussian field specified by its mean function, *μ* = *μ*(*Y*(*s*; *t*)) and the spatiotemporal covariance function $\Sigma =Cov(Y\left(s;t\right),Y\left(s^{{}^{\prime }};t^{{}^{\prime }}\right))=\sigma^{2}C(\left(s;t\right),\left(s^{{}^{\prime }};t^{{}^{\prime }}\right))$ with (*s*; *t*), (*s*′; *t*′) ∈ R^2^ × R. The spatiotemporal process is second-order stationary if its mean is constant and the spatiotemporal covariance function depends on the locations and time points only through the spatial distance vector *d* = *s* − *s*′ ∈ R^2^ and the temporal lag *l* = *t* − *t*′ ∈ R [8]. The covariance function is isotropic and stationary, the covariance function is stationary only a function of a relative position of two locations and isotropic a covariance function depends only on the euclidian distance between two locations. Since a covariance matrix is positive definite, the spatiotemporal covariance function must be a positive definite function. Although Gaussian fields are expedient from both an analytical and a practical approach, the computational issues have always been complex. Although Gaussian fields are expedient from both an analytical and a practical approach, the computational issues have always been complex. This is due to the general computational cost of O(n^3^) to factorize the dense *n* × *n* spatiotemporal covariance matrices. to overcome ‘the big n problem’, by doing exact computations on a simplified Gaussian model of low rank [8], [12], [13], applied covariance tapering to zero-out parts of the covariance matrix to improve a computational speed. However, the sparsity pattern will depend on the range of the GFs [14], thus to avoid this complexity the GF is substituted by a Gaussian Markov random field (GMRF). The GMRF illustration can be constructed plainly by using a certain stochastic partial differential equation (SPDE) [8] which has GFs with Matern covariance function as the solution when driven by Gaussian white noise. The result is a basis function representation with piecewise linear basis functions and Gaussian weights with Markov dependences determined by a general triangulation of the domain. The critical lump is that the spatiotemporal covariance function and the dense covariance the matrix of a GF are substituted, respectively, by a neighborhood structure and by a sparse precision matrix that together defines a GMRF. Indeed, GMRFs are defined by a precision matrix with a sparse structure for which it is possible to use computationally effective numerical methods, especially for fast matrix factorization [14]. Moreover, when working with Bayesian inference for GMRFs, it is thinkable to make use of the Integrated Nested Laplace Approximation (INLA) procedure proposed by [15] as an alternative to MCMC methods for latent Gaussian field models. The best marvelous edge of INLA is computational because it produces almost immediately precise approximations to posterior distributions, also in the case of complex models. Thus, the joint use of the SPDE approach together with the INLA method is a candidate for being a powerful solution in overcoming the computational issues related to GF modeling. The main aim of this paper is the spatiotemporal modeling of the infected rate of visceral leishmaniasis in human (VLH) over a stochastic partial differential equation (SPDE) approach using Integrated nested Laplace approximations (INLA) technique.

$$\begin{array}{c} Y(s;t)=\beta Z(s,t)+\Phi (s,t)+\epsilon (s,t) \end{array}$$ (2)

$$\begin{array}{c} \Phi (s,t)=\lambda \Phi (s,t-1)+\gamma (s,t) \end{array}$$ (3)

The spatiotemporal model above defines a hierarchical model described by a Gaussian field (GF), *Y*(*s*; *t*) assembled from external covariate information *Z*(*s*, *t*), microscale spatiotemporal variation, *ϵ*(*s*, *t*) and a first-order autoregressive a dynamic model for the latent process Φ(*s*, *t*) with spatially correlated innovations *γ*(*s*, *t*). This kind of model has extensively applied in the diseases infection and disease modeling literature appreciations towards its suppleness in modeling the effect of relevant external variables (i.e. meteorological, sociodemographic and ecological variables) as well as time and space dependence. Thus, spatiotemporal modeling of the infected rate of visceral leishmaniasis in human in Amhara regional state, Ethiopia, using weekly data from first week of January 2015 to last week of December 2017 with a total of 156 weeks of 10 different locations in the area a total of 1560 spatiotemporal data for estimation and we used 5 validation location with a total of 780 spatiotemporal data.

## Materials and methods

The main aim of this study is to identify the determinates of the infected rate of visceral leishmaniasis in Humans by using dynamic spatiotemporal modelling.

### Hierarchical spatiotemporal modeling of the infected rate of visceral leishmaniasis(VLH)

Leishmaniases are an assembly of diseases triggered by more than 20 different species of the protozoan genus Leishmania that is spread between a human being and other mammalian hosts by phlebotomine sandflies [7], [16]. Visceral leishmaniasis (VL), which affects interior body part, for instance, the liver and spleen, if it is not identified and cured in the preliminary phases, it usually leads towards death [17]. The infection endangers about 350 million persons in 98 countries, most of them in the poorer regions of the globe [1], [6]. In Ethiopia, an estimated over 2500 to 4000 new VL cases happen per annum and over 3.2 million persons are at threat of infection of VL [16], [18]. It widely spread over the arid and semi-arid parts of the country. Despite the symptom of visceral leishmaniasis in human (VLH) is fundamentally similar to malaria and enteric fever, reliable laboratory methods become mandatory for the accurate diagnosis of the infection.VL in Ethiopia is caused by L. donovani with an anthroponotic transmission. Leishmania infections do not always equate with clinical illness. The ratio of incident asymptomatic infections to incident clinical cases varies among geographic regions. Most infections in immuno-competent individuals remain asymptomatic [19]. In addition, untreated individuals might act as reservoirs putting the community at risk of ongoing transmissions.

**Fig 2:**
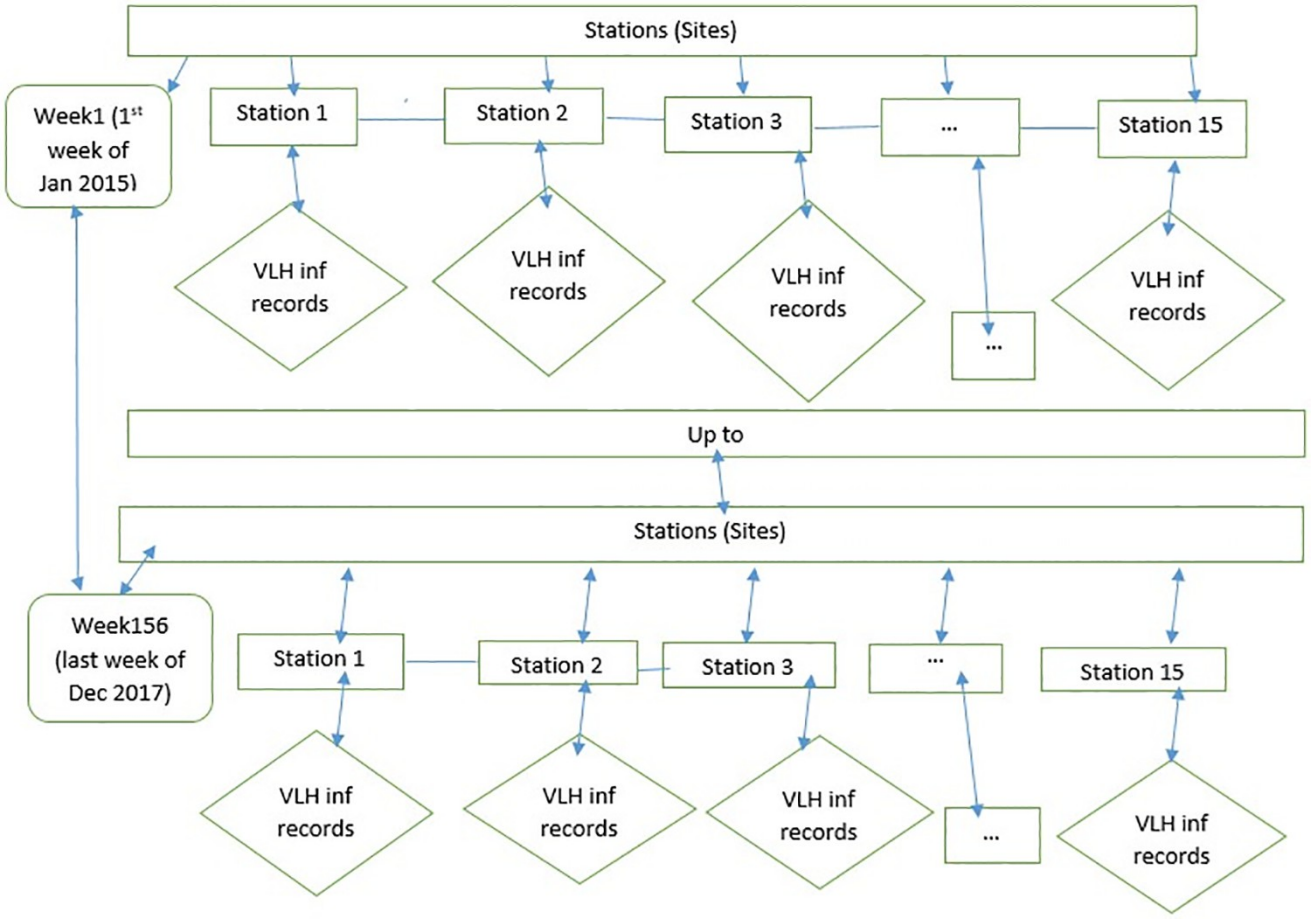
The flowchart shows that weekly visceral leishmaniasis in human (VLH) infection at every 15 sites (stations).

**Fig 3:**
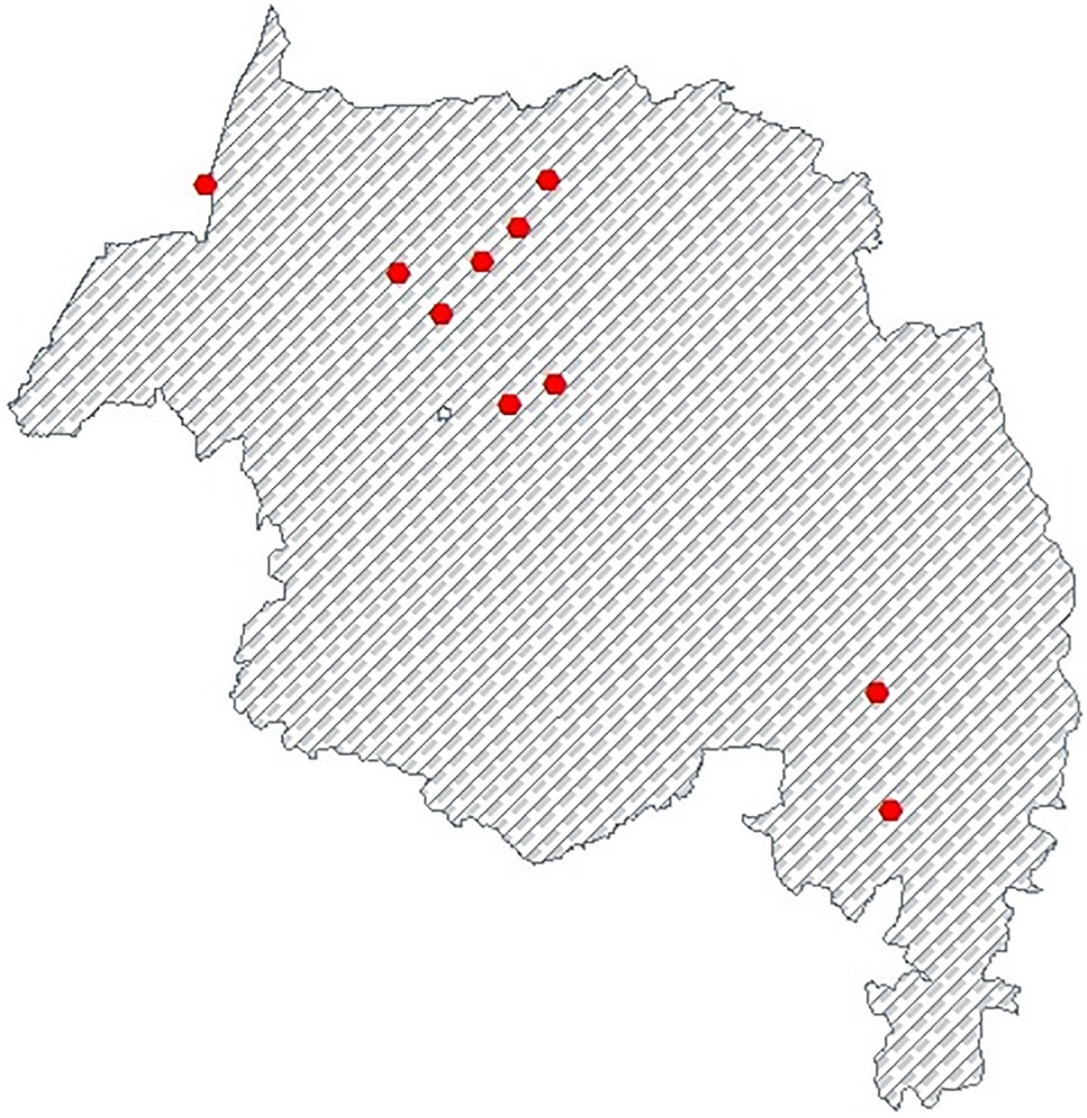
The map shows 10 locations of VLH infection rate stations.

**Fig 4:**
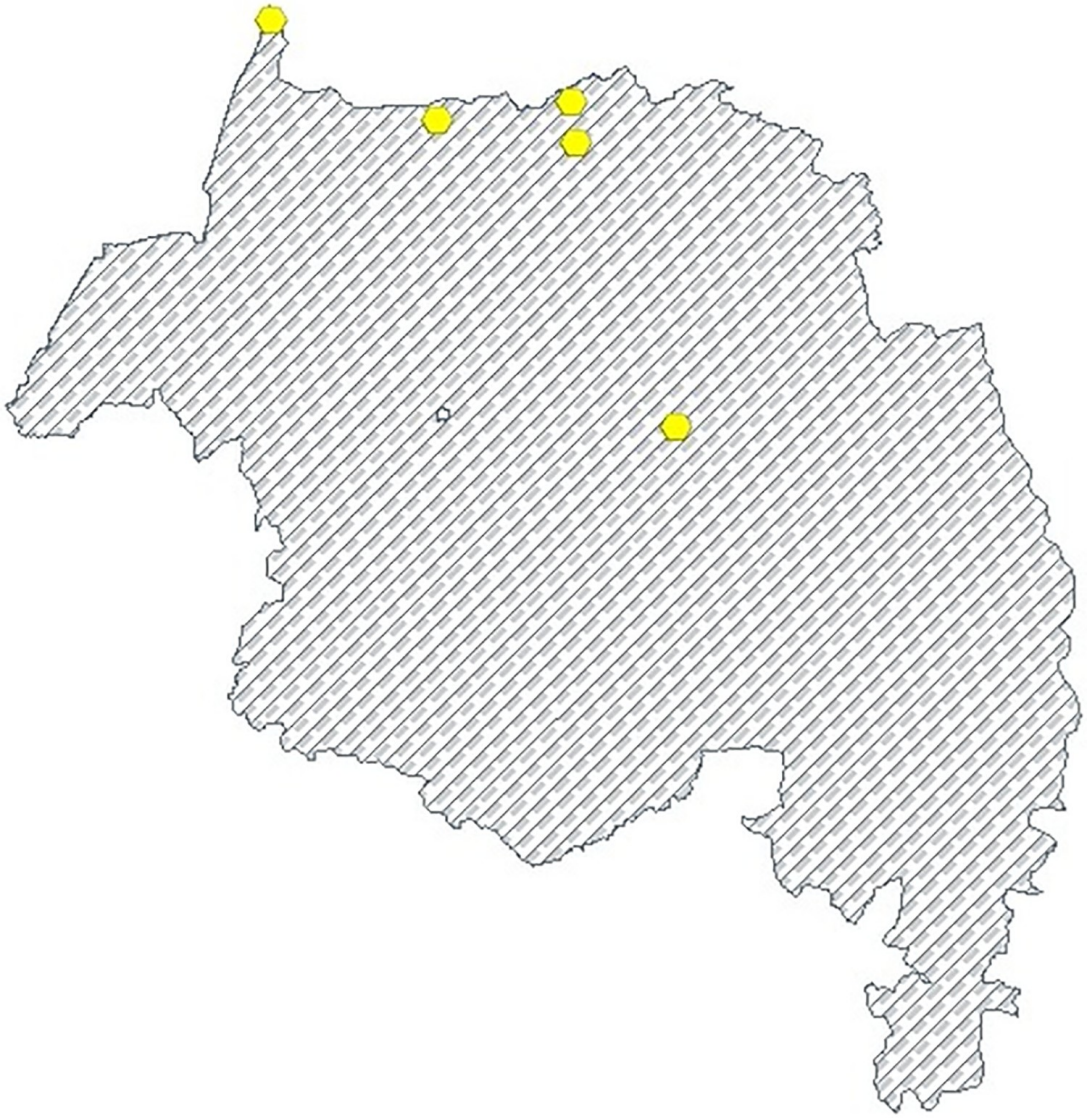
The map shows 5 locations of VLH infection rate validation stations.

Fig 2 below shows that the flowchart of VLH infection from the first week of January 2015(week 1) to the last week of December 2017(week 156) at every 15 stations. We collect the records of VLH infection in a spatiotemporal manner by considering all of 15 stations in the endemic regions. Furthermore, we divided these stations into estimation and validation sites, by selecting 10 of stations for estimations and the remaining 5 stations for validation in a random fashion. In this study, VLH infection was tested at each treatment site in the endemic area of Amhara regional state, based on the symptom of the patient and its traveling history during admission. Visceral Leishmaniasis should always be suspected when an individual presents with prolonged fever from the endemic areas. The test of VLH infection has been depending on clinical, serological, parasitological and molecular tests to detect the infection based on the species of leishmaniasis. We collected data at every 15 VLH stations in the endemic regions, from VLH infection registration book weekly spatiotemporal data from January 2015 to December 2017 and we calculated the weekly infection rate based on the total number of infected population and the total number of populations resident in the region. Thus, ministry heath has to access infected rate of VLH in order to take appropriate and operative actions in order to refine the situation of the most VLH infection regions and to get the map of the infection rate of VLH. As a result, we propose a hierarchical spatiotemporal model able to catch the complex spatiotemporal dynamics of infection the rate of VLH, including the meteorological, sociodemographic and geographical variables as external covariates. We consider Amhara Regional State, Ethiopia. We analyzed a weekly average infected rate of visceral leishmaniasis in human (VLH) particularly in South and North Gondar zone of Amhara region by considering infection areas from the first week of January 2015 to last week of December 2017 with a total of T = 156 weeks. In particular, we consider a total of m = 10 stations for spatiotemporal estimation as shown in Fig 3 below the red hexagon indicates the location of 10 stations and in Fig 4 below the yellow hexagon indicates the location of 5 validation stations. In addition, we have used external covariates such as meteorological, sociodemographic and geographical variables. Moreover, we consider altitude (A, m) and spatial geographic coordinates (X and Y, in km) in addition to weekly temporal dynamics. The data for this study is provided by the University of Gondar leishmaniasis research and treatment center, Ethiopian Central Statistics Agency(CSA) and Amhara Regional Health Bureau.

### Spatiotemporal models

We assumed that *Y*(*s*; *t*) be the realization of the spatiotemporal process *Y*(, ; ,) that indicates the weekly average infected rate of Visceral leishmaniasis in human(VLH) at locations *i* = 1, 2, …, *m* located at *s*_*i*_ stations and week *t* = 1, 2, …, *T*, We assumed the following spatiotemporal model;

$$\begin{array}{c} Y\left(s_{i};t\right)=\beta Z\left(s_{i},t\right)+\Phi \left(s_{i},t\right)+\epsilon \left(s_{i},t\right) \end{array}$$ (4)

Where; *Z*(*s*_*i*_, *t*) = *Z*_1_(*s*_*i*_, *t*), …, *Z*_*p*_(*s*_*i*_, *t*) denote the vector of covariates and *β* = (*β*_1_, *β*_2_, …, *β*_*p*_)′ is a coefficient of the vector, Moreover; $\epsilon \left(s_{i},t\right)∼N(0,\sigma_{\epsilon }^{2})$ is spatiotemporal Gaussian white noise process both serially and spatially uncorrelated. *ϵ*(*s*_*i*_, *t*) is the realization of state process of the unobserved rate of infection of VLH. We assumed the spatiotemporal Gaussian field process that changes in time with first-order autoregressive dynamics with a coefficient of λ and colored innovations is given by;

$$\begin{array}{c} \Phi \left(s_{i},t\right)=\lambda \Phi \left(s_{i},t-1\right)+\gamma \left(s_{i},t\right) \end{array}$$ (5)

For *t* = 2, 3, …, *T* where |λ| < 1 and Φ(*s*_*i*_, 1) is derived from a stationary Gaussian distribution $∼N(0,\frac{\sigma_{\gamma }^{2}}{1-\lambda^{2}})$ Moreover, *γ*(*s*_*i*_, *t*) has a zero-mean Gaussian distribution, is assumed to be temporally independent and characterized by spatiotemporal covariance functions;

$$\begin{array}{c} Cov(\gamma \left(s_{i},t\right),\gamma \left(s_{i}^{{}^{\prime }},t^{{}^{\prime }}\right))=\{\begin{array}{cc} 0,if & t\neq t^{{}^{\prime }}\\ \sigma_{\gamma }^{2}\left(r\right) & ift=t^{{}^{\prime }} \end{array} \end{array}$$

for *i* ≠ *j* the purely spatial correlation function *C*(*r*) is depending on the location *s*_*i*_ and *s*_*j*_ only through the euclidian spatial distance *r* = ||*s*_*i*_ − *s*_*j*_|| ∈ R. Thus, the process is second order stationary and isotropic [10]. It follows that $Var\left(\gamma \left(s_{i},t\right)\right)=\sigma_{\gamma }^{2}$ for each *s*_*i*_ and *t*. The Spatial correlation function is defined as a Matern function;

$$\begin{array}{c} C\left(r\right)=\frac{1}{\Gamma \left(v\right)2^{v-1}}{\left(kr\right)}^{v}\kappa_{v}\left(kr\right) \end{array}$$

With *κ*_*v*_ denoting the modified Bessel function of the second kind and order *v* > 0, The parameter *v*, which usually kept stable measures of the degree of smoothness of the process and its integer value decides the mean square differentiability of the process, *k* > 0 is the scaling parameter related to the range *ρ* we use empirically derived definition $\rho =\frac{\sqrt{8v}}{k}$ with *ρ* corresponding the distance where the spatial correlation is close to 0.1 [8], [20]. From Eqs (4) and (5) by collecting observation in vector *t* we have:

$$\begin{array}{l} \;Y_{t}={\left(y\left(s_{1},t\right),y\left(s_{2},t\right),\ldots ,y\left(s_{m},t\right)\right)}^{{}^{\prime }}\\ \;Z_{t}={\left(z\left(s_{1},t\right),z\left(s_{2},t\right),\ldots ,z\left(s_{m},t\right)\right)}^{{}^{\prime }}\\ \Phi_{t}={\left(\Phi \left(s_{1},t\right),\Phi \left(s_{2},t\right),\ldots ,\Phi \left(s_{m},t\right)\right)}^{{}^{\prime }}\\ \;\epsilon_{t}={\left(\epsilon \left(s_{1},t\right),\epsilon \left(s_{2},t\right),\ldots ,\epsilon \left(s_{m},t\right)\right)}^{{}^{\prime }} \end{array}$$

It follows that;

$$\begin{array}{c} Y_{t}=\beta Z_{t}+\Phi_{t}+\epsilon_{t} \end{array}$$ (6)

$\epsilon_{t}∼N(0,\sigma_{\epsilon }^{2}I_{m})$ and also;

$$\begin{array}{c} \Phi_{t}=\lambda \Phi_{t-1}+\gamma_{t} \end{array}$$ (7)

$\gamma_{t}∼N(0,\Sigma =\sigma_{\epsilon }^{2}\tilde{\Sigma })$, we assume is coming from the stationary distribution of AR(1) process with, $\Phi_{1}∼N(0,\frac{\Sigma }{1-\lambda^{2}})=∼N(0,\frac{\sigma_{\epsilon }^{2}\tilde{\Sigma }}{1-\lambda^{2}})$ Moreover, the dense correlation matrix of dimension *m* with an element *C*(||*s*_*i*_ − *s*_*j*_||), Where *C*(*r*) is a Matern covariance function;

$$\begin{array}{c} C\left(r\right)=\frac{1}{\Gamma \left(v\right)2^{v-1}}{\left(kr\right)}^{v}\kappa_{v}\left(kr\right) \end{array}$$

and parameterized by *κ* and *v*. Let $\Theta =\left(\beta ,\sigma_{\epsilon }^{2},\lambda ,\sigma_{\gamma }^{2},\kappa \right)$ denote the parameter of the vector to be estimated, thus, the joint posterior distribution is given by;

$$\begin{array}{c} P(\Theta ,\Phi |Y)\propto P(Y|\Theta ,\Phi )P(\Phi |\Theta )P(\Theta ) \end{array}$$ (8)

Where *P*(.) denotes the probability density functions, *Y* = (*Y*_*t*_) and Φ = (Φ_*t*_) with *t* = 2, 3, …, *T* and assume the hyper parameters are independent $P(\Theta )=\prod_{i=1}^{dim(\Theta )}P\left(\Theta_{i}\right)$, The left-hand side of Eq (8) above is the joint posterior distribution and the right-hand side of Eq (8), *P*(*Y*|Θ, Φ) is the data model (likelihood of the data), *P*(Φ|Θ) is the latent (unobserved) process model and *P*(Θ) is the parameter model. From Eq (6) above we have

$$\begin{array}{c} Y_{t}=\beta Z_{t}+\Phi_{t}+\epsilon_{t} \end{array}$$

$\epsilon_{t}∼N(0,\sigma_{\epsilon }^{2}I_{m})$ Thus; the data model (likelihood of the data) can be written as

$$\begin{array}{c} P(Y_{t}|\Phi ,\Theta )∼N(\beta Z_{t}+\Phi_{t},\sigma_{\epsilon }^{2}I_{m})\\ ={\left(\frac{1}{\sqrt{2\pi }\sigma_{\epsilon }}\right)}^{mT}exp^{\frac{-1}{2}}\sum_{i=1}^{T}(\frac{\epsilon_{t}\epsilon_{t}^{{}^{\prime }}}{\sigma_{\epsilon }^{2}}) \end{array}$$

but *ϵ*_*t*_ = *Y*_*t*_ − *βZ*_*t*_ − Φ_*t*_

$$\begin{array}{c} P(Y_{t}|\Phi ,\Theta )\propto {\left(\sigma_{\epsilon }^{2}\right)}^{\frac{-mT}{2}}exp^{\frac{-1}{2\sigma_{\epsilon }^{2}}}[\sum_{i=1}^{T}(\left(Y_{t}-\beta Z_{t}-\Phi_{t}\right){\left(Y_{t}-\beta Z_{t}-\Phi_{t}\right)}^{{}^{\prime }})] \end{array}$$ (9)

The joint distribution of spatiotemporal process model is can be factorized as with the total law of probability and Markovian process;

$$\begin{array}{c} P(\Phi_{1},\Phi_{2},\ldots ,\Phi_{T}|\Theta )=P(\Phi_{T}|\Phi_{T-1},\Phi_{T-2},\ldots ,\Phi_{1},\Theta )\times \ldots ,P(\Phi_{2}|\Phi_{1},\Theta )\times P(\Phi_{1}|\Theta ) \end{array}$$

using a Markovian process assumption we have;

$$\begin{array}{c} P(\Phi_{T}|\Phi_{T-1},\Phi_{T-2},\ldots ,\Phi_{1},\Theta )=P(\Phi_{T}|\Phi_{T-1},\Theta ) \end{array}$$

We have the joint distribution of the process model;

$$\begin{array}{c} P(\Phi_{1},\Phi_{2},\ldots ,\Phi_{T}|\Theta )=P(\Phi_{1}|\Theta )\prod_{t=2}^{T}P(\Phi_{t}|\Phi_{t-1},\Theta ) \end{array}$$ (10)

We assume the hyperparameter are independent, the parameter model is given by;

$$\begin{array}{c} P(\Theta )=\prod_{i=1}^{dim(\Theta )}P\left(\Theta_{i}\right) \end{array}$$ (11)

The posterior distribution is given by the product of the data model, process model, and parameter model [21]. we assume Φ_1_ is AR(1) stationary process, $\Phi_{1}|\Theta ∼N(0,\frac{\Sigma }{1-\lambda^{2}})$, Φ_1_|Θ also called the initial distribution [21].

$$\begin{array}{c} P(\Phi_{1}|\Theta )={\left(\frac{1}{\sqrt{2\pi }{|\frac{\Sigma }{1-\lambda^{2}}|}^{\frac{1}{2}}}\right)}^{m}exp^{\frac{-1}{2}}(\Phi_{1}^{{}^{\prime }}{\left(\frac{\Sigma }{1-\lambda^{2}}\right)}^{-1}\Phi_{1}) \end{array}$$

From above we define and assume $\Sigma =\sigma_{\gamma }^{2}\tilde{\Sigma }$

$$\begin{array}{c} P(\Phi_{1}|\Theta )\propto {\left(\frac{\sigma_{\gamma }^{2}}{1-\lambda^{2}}\right)}^{\frac{-m}{2}}{|\tilde{\Sigma }|}^{\frac{-1}{2}}exp^{\left(-\frac{1-\lambda^{2}}{2\sigma_{\gamma }^{2}}\Phi_{1}^{{}^{\prime }}{\tilde{\Sigma }}^{-1}\Phi_{1}\right)} \end{array}$$ (12)

Also the evolution distribution; *P*(Φ_*t*_|Φ_*t*−1_, Θ) since follows an AR(1) process, Φ_*t*_ = λΦ_*t*−1_ + *γ*_*t*_ and $\gamma_{t}∼N(0,\Sigma =\sigma_{\epsilon }^{2}\tilde{\Sigma })$, The joint eveloution distribution is given as;

$$\begin{array}{c} P(\Phi_{t}|\Phi_{t-1},\Theta )={\left(\frac{1}{\sqrt{2\pi \sigma_{\gamma }^{2}}}\right)}^{m(T-1)}{|\tilde{\Sigma }|}^{-(\frac{T-1}{2})}exp^{\left(-\frac{1}{2\sigma_{\gamma }^{2}}\sum_{i=1}^{T}\left(\Phi_{t}-\lambda \Phi_{t-1}\right){\tilde{\Sigma }}^{-1}{\left(\Phi_{t}-\lambda \Phi_{t-1}\right)}^{\prime }\right)} \end{array}$$

$$\begin{array}{c} P\left(\Phi_{t}|\Phi_{t-1},\Theta \right)\propto {\left(\sigma_{\gamma }^{2}\right)}^{\frac{-m\left(T-1\right)}{2}}|\tilde{\Sigma }{|}^{-\left(\frac{T-1}{2}\right)}exp^{\left(-\frac{1}{2\sigma_{\gamma }^{2}}\sum_{i=1}^{T}\left(\Phi_{t}-\lambda \Phi_{t-1}\right){\tilde{\Sigma }}^{-1}\left(\Phi_{t}-\lambda \Phi_{t-1}\right)\prime \right)} \end{array}$$ (13)

the joint process model is expressed as the product of the joint initial distribution and the joint evolution distribution [21], Thus from Eqs (12) and (13), we have the process model the joint distribution given by;

$$\begin{array}{c} P(\Phi_{1},\Phi_{2},\ldots ,\Phi_{T}|\Theta )=P(\Phi_{1}|\Theta )\prod_{t=2}^{T}P(\Phi_{t}|\Phi_{t-1},\Theta ) \end{array}$$

$$\begin{array}{c} \begin{array}{cc} \propto {\left(\frac{\sigma_{\gamma }^{2}}{1-\lambda^{2}}\right)}^{\frac{-m}{2}}{|\tilde{\Sigma }|}^{\frac{-1}{2}}exp^{\left(-\frac{1-\lambda^{2}}{2\sigma_{\gamma }^{2}}\Phi_{1}^{{}^{\prime }}{\tilde{\Sigma }}^{-1}\Phi_{1}\right)}\times \\ & {\left(\sigma_{\gamma }^{2}\right)}^{\frac{-m(T-1)}{2}}{|\tilde{\Sigma }|}^{-(\frac{T-1}{2})}\times \\ & exp^{\left(-\frac{1}{2\sigma_{\gamma }^{2}}\sum_{i=1}^{T}\left(\Phi_{t}-\lambda \Phi_{t-1}\right){\tilde{\Sigma }}^{-1}{\left(\Phi_{t}-\lambda \Phi_{t-1}\right)}^{{}^{\prime }}\right)} \end{array} \end{array}$$ (14)

The parameter model also from Eq (11) above;

$$\begin{array}{c} P(\Theta )=\prod_{i=1}^{dim(\Theta )}P\left(\Theta_{i}\right) \end{array}$$

Thus, the joint posterior model is the product of the data model, process model and parameter model [21] give us;

$$\begin{array}{c} A={\left(\sigma_{\epsilon }^{2}\right)}^{\frac{-mT}{2}}exp^{\frac{-1}{2\sigma_{\epsilon }^{2}}}[\sum_{i=1}^{T}(\left(Y_{t}-\beta Z_{t}-\Phi_{t}\right){\left(Y_{t}-\beta Z_{t}-\Phi_{t}\right)}^{{}^{\prime }})] \end{array}$$ (15)

$$\begin{array}{c} \begin{array}{cc} B={\left(\frac{\sigma_{\gamma }^{2}}{1-\lambda^{2}}\right)}^{\frac{-m}{2}}{|\tilde{\Sigma }|}^{\frac{-1}{2}}exp^{\left(-\frac{1-\lambda^{2}}{2\sigma_{\gamma }^{2}}\Phi_{1}^{{}^{\prime }}{\tilde{\Sigma }}^{-1}\Phi_{1}\right)}\times \\ & {\left(\sigma_{\gamma }^{2}\right)}^{\frac{-m(T-1)}{2}}{|\tilde{\Sigma }|}^{-(\frac{T-1}{2})}\times \\ & exp^{\left(-\frac{1}{2\sigma_{\gamma }^{2}}\sum_{i=1}^{T}\left(\Phi_{t}-\lambda \Phi_{t-1}\right){\tilde{\Sigma }}^{-1}{\left(\Phi_{t}-\lambda \Phi_{t-1}\right)}^{{}^{\prime }}\right)} \end{array} \end{array}$$ (16)

$$\begin{array}{c} C=\prod_{i=1}^{dim(\Theta )}P\left(\Theta_{i}\right) \end{array}$$ (17)

The joint posterior distribution;

$$\begin{array}{c} P(\Theta ,\Phi |Y)\propto P(Y|\Theta ,\Phi )P(\Phi |\Theta )P(\Theta ) \end{array}$$

From Eqs (15) to (17) the joint posterior distribution is approximated by;

$$\begin{array}{c} P(\Theta ,\Phi |Y)\propto A\times B\times C \end{array}$$ (18)

where $|\tilde{\Sigma }|$ is the determinant of the dense m-dimensional correlation matrix $\tilde{\Sigma }$.

### GMRF and Stochastics Partial Differential Equation (SPDE) approach

#### Gaussian Markov random field (GMRF)

GRMF is a zero mean multivariate Gaussian distribution with a sparse precision matrix [8], [20].

$$\begin{array}{c} P(X)={\left(\frac{1}{\sqrt{2\pi }}\right)}^{n}{\left|Q\right|}^{\frac{1}{2}}exp^{\frac{-1}{2}(X^{{}^{\prime }}QX)} \end{array}$$

Where *Q* is the precision matrix it just the inverse of the covariance matrix.

**Theorem**: *Q*_*ij*_ = 0 ⇔ *X*_*i*_ is conditionally independent *X*_*j*_ given all other variables, *i*.*e* (*X*_*i*_ ⊥ *X*_*j*_|*X*_−*ij*_). A collection of a random variable *X* is a Gaussian Markov random field (GMRF) with respect to a graph with vertex *V* and edge *G*, *i*.*e* *G* = (*V*, *E*) with mean *μ* and a precision matrix *Q* with its probability distribution of a multivariate Gaussian, if the elements of the precision matrix is non zero (*Q*_*ij*_ ≠ 0) implies that (*i*, *j*) ∈ *E* ∀*i* ≠ *j*.

$$\begin{array}{c} P(X)={\left(\frac{1}{\sqrt{2\pi }}\right)}^{n}{\left|Q\right|}^{\frac{1}{2}}exp^{\frac{-1}{2}({\left(X-\mu \right)}^{{}^{\prime }}Q\left(X-\mu \right))} \end{array}$$ (19)

A GMRF is a spatial process that used to model the spatial dependence of data observed on areal units, for instance, regular grid, lattice structure or geographical regions [14]. A Gaussian Markov random field *X* can be specified over the conditional distributions for every part given all the others. Furthermore, the Markovian property is connected to the definition of a neighborhood structure, in that the full conditional distribution of *X*_*i*_ for *i* = 1, 2, 3 …, *n* depends only on a few of the components of *X*. This set of components is denoted by *ζ*_*i*_, which constitutes the set of neighbors of unit *i*, and

$$\begin{array}{c} P(X_{i}|X_{-}i)=P(X_{i}|X_{\zeta_{i}}) \end{array}$$ (20)

where the notation *X*_−*i*_ denotes all elements in *X* except for *X*_*i*_. This implies that given the neighborhood *ζ*_*i*_, the terms *X*_*i*_ and *X*_−(*i*,*ζ*_*i*_)_ are independent. The conditional independence relation can be written as;

$$\begin{array}{c} X_{i}⊥X_{-(i,\zeta_{i})}|X_{\zeta_{i}} \end{array}$$

for *i* = 1, …, *n*. The main aims of this conditional independence property are rigorously related to the precision matrix *Q*, Generally for *i* and *j* with *i* ≠ *j* it holds that;

$$\begin{array}{c} X_{i}⊥X_{j}|X_{-}\left(i,j\right)\Leftrightarrow Q_{ij}=0 \end{array}$$

which indicates that the nonzero pattern of *Q* is given by the neighborhood structure of the process. Thus, *Q*_*ij*_ ≠ 0 if *j* ∈ (*i*, *ζ*_*i*_).

The computational advantage of making inference with a GMRF stalks directly from the sparsity of the precision matrix *Q*. The computational properties of GMRFs are enhanced by utilizing Integrated Nested Laplace Approximations (INLA) for Bayesian inference [9], [15]. is a computationally capable strategy that produces quick and exact approximations to posterior distributions.

### Stochastics Partial Differential Equation (SPDE) approach

Let *X*(*s*) ≡ (*x*(*s*), *s* ∈ D_*s*_ ⊆ R^2^) denote a Matern field, this implies that the second order stationary and isotropic Gaussian field with Materns covariance functions

$$\begin{array}{c} C\left(r\right)=\frac{1}{\Gamma \left(v\right)2^{v-1}}{\left(kr\right)}^{v}\kappa_{v}\left(kr\right) \end{array}$$

and depending on the scale *κ* and smoothness parameter *v*. Suppose we observe a realization of the spatial process *X*(*s*_*i*_) at *m* spatial location *s*_1_, *s*_2_, …, *s*_*m*_. The primary points of the SPDE approach are to discover a GMRF, with a nearby neighborhood and a sparse precision matrix *Q*, that profoundly speaks to the Matern field. Accordingly, it is conceivable to make inference utilizing the GMRF with the best computational expenses. This makes it conceivable to maintain a computational problem-related to the large dimension of the matrix [8] that emerges when working with the dense covariance matrix of a GF.

On a very basic level, the SPDE approach utilizes a finite element representation to define the Matrn field as a linear combination of basis functions defined on a triangulation of the domain *D*_*s*_. This comprises of subdividing *D*_*s*_ into a set of non-crossing triangles meeting in at most a typical edge or corner. To begin with, the triangle initial vertices are put at the areas *s*_1_, *s*_2_, …, *s*_*m*_ and after that extra vertices are included request to get an appropriate triangulation valuable for spatial forecast purposes. Matern covariance functions show up in different fields [8] in any case, essential acknowledgment we will make utilization of its that GF *X*(*s*) with Matern covariance is a solution to the linear fractional SPDE

$$\begin{array}{c} {\left(\kappa^{2}-\Delta \right)}^{\frac{\alpha }{2}}X\left(s\right)=\omega \left(s\right) \end{array}$$ (21)

*s* ∈ R^*m*^, $\alpha =v+\frac{m}{2}$, *κ* > 0, *v* > 0, where ${\left(\kappa^{2}-\Delta \right)}^{\frac{\alpha }{2}}$ is a pseudodifferential operator. The innovation process *ω* is spatial Gaussian white noise with unit variance and Δ is the Laplacian

$$\begin{array}{c} \Delta =\sum_{i=1}^{m}\frac{\partial^{2}}{\partial x_{i}^{2}} \end{array}$$

and the marginal variance

$$\begin{array}{c} \sigma^{2}=\frac{\Gamma (v)}{\Gamma (v+\frac{m}{2})+4\pi^{\frac{m}{2}}\kappa^{2v}} \end{array}$$

We shall name any solution to Eq (21) is a Matern field [8]. The Matern field is the main stationary solution for SPDE. Ordinarily, the triangulation has amplified the base inside the triangle edge, purported Delaunay triangulations, which guarantees that the advances among little and substantial triangles are smooth. The additional vertices are included heuristically [8], [20] to endeavor to limit the aggregate number of triangles that are expected to satisfy the size and shape constraints. To build a GMRF portrayal of Matern’s field on the triangulated grid, we begin the stochastics week solution of SPDE in Eq (21). We define the inner product;

$$\begin{array}{c} (f,g)=\int f\left(s\right)g\left(s\right)ds \end{array}$$

where the integral is over the region of interest. The stochastic weak solution of the SPDE is found by requiring that

$$\begin{array}{c} \left(\langle \eta_{j},{\left(\kappa^{2}-\Delta \right)}^{\frac{\alpha }{2}}x\rangle ,j=1,2,3\ldots ,q)\underline{Dist}(\langle \eta_{j},\omega \rangle ,j=1,2,3\ldots ,q\right) \end{array}$$

For every appropriate finite set of the test function (*η*_*j*_, *j* = 1, 2, 3…, *q*). Given the triangulation, the basis function representation of the Matérn field, The finite element representation the solution to SPDE [8], [22].

$$\begin{array}{c} X\left(S\right)=\sum_{h=1}^{k}\psi_{h}\left(S\right)\omega_{h} \end{array}$$ (22)

for some chosen basis functions *ψ*_*h*_ and Gaussian-distributed weights *ω*_*h*_. Here, *n* is the number of vertices in the triangulation. We choose to use functions *ψ*_*h*_ that are piecewise linear in each triangle, defined such that *ψ*_*h*_ is 1 at vertex *h* and 0 at all other vertices. The key purpose of the SPDE approach is the finite element representation in Eq (22) that sets up the connection between the GF *X*(*s*) and the GMRF characterized by the Gaussian weights (*ω*_*h*_) to which a Markovian structure can be given.

Specifically, the precision matrix *Q* of the GMRF *ω* = (*ω*_1_, *ω*_2_, …, *ω*_*n*_) is a function of *κ*^2^ [8], for *α* = 1, 2, 3, … and *v* = 0, 1, 2, … with *α* = *v* + 1. This characterizes an explicit mapping from the parameters of the GF covariance function (*κ*) and (*v*) to the elements of the precision matrix *Q* of the GMRF *ω*, with a computational expense of *O*(*n*) for any triangulation [20]. We describe how to actualize the spatiotemporal model characterized in Eqs (4) and (5) by utilizing the SPDE approach. We focus on reclassifying the model make use of the connection between GF and GMRF and estimation of the posterior parameters.

For each time point *t* = 1, 2, 3…, *T*, the Matern field *γ*_*t*_ in Eq (7) is characterized through GMRF, ${\tilde{\gamma }}_{t}∼N\left(0,Q_{s}^{-1}\right)$ where, Q_*s*_ is the precision matrix originates from the SPDE approach. The matrix Q_*s*_ does not change in time because of the serial independence assumption of Eq (5) and its dimension *n* is given by the number of vertices of the domain triangulation. In this manner Eq (7) can modify as;

$$\begin{array}{c} \Phi_{t}=\lambda \Phi_{t-1}+\tilde{\gamma_{t}} \end{array}$$ (23)

$$\begin{array}{c} \tilde{\gamma_{t}}∼N(0,Q_{s}^{-1}) \end{array}$$

for *t* = 1, 2, 3…, *T* with $\Phi_{1}∼N(0,\frac{Q_{s}^{-1}}{1-\lambda^{2}})$ follows that the joint distribution of *Tn* dimensional GMRF, $\Phi =(\Phi_{1}^{{}^{\prime }},\Phi_{2}^{{}^{\prime }},\ldots ,\Phi_{T}^{{}^{\prime }})$ is given by;

$$\begin{array}{c} \Phi ∼N(0,Q^{-1}) \end{array}$$ (24)

Where, Q = Q_*s*_ ⊗ Q_*T*_ and Q_*T*_ is *T*- dimensional precession matrix of the autoregressive process of order 1 in Eq (23). Equation Eq (6) we can rewrite as also;

$$\begin{array}{c} Y_{t}=\beta Z_{t}+H\Phi_{t}+\epsilon_{t},\epsilon_{t}∼N(0,\sigma_{\epsilon }^{2}I_{m}) \end{array}$$ (25)

where the (*m* × *n*) dimensional matrix H selects the value of the GMRF Φ_*t*_ for each observation vector *Y*_*t*_. In particular, H is a sparse matrix with only one unit element for each row and such that;

$$\begin{array}{c} Y(s_{i},t)=\beta Z(s_{i},t)+\sum_{i=1}^{n}H_{ij}\Phi_{t}+\epsilon (s_{i},t) \end{array}$$

Where H_*ij*_ = 1 if the triangulation vector *j* is placed at location *s*_*i*_ and 0 otherwise.

### Parameter estimation

The hierarchical model characterized by Eqs (23) and (25) belongs to the class of latent Gaussian models and can be evaluated using the INLA procedure proposed by [15]. INLA is a computational methodology for Bayesian inference and is an option to MCMC for getting the approximated posterior marginals for the latent variable and additionally for the hyper parameters.

Let *X* = (Φ, *β*) mean the basic latent field with a priori independent parts. We dole out a vague Gaussian with known precession to *β* and the Gaussian Markov Random Field (GMRF) distribution to Φ in Eq (24). Consequently the density *P*(*X*|Θ) is a Gaussian distribution with mean zero and precession matrix *Q* with hyperparameter vector $\left(\sigma_{\gamma }^{2},\lambda ,\kappa \right)$. Also, we have the observation *Y* = (*Y*_*t*_) is normally distributed and conditionally independent given *X* and $\sigma_{\epsilon }^{2}$. Thus, $\Theta =(\sigma_{\gamma }^{2},\sigma_{\epsilon }^{2},\lambda ,\kappa )$ is the hyper parameter vector, The joint posterior distribution is given by;

$$\begin{array}{c} P(X,\Theta |Y)=P\left(\Theta \right)P\left(X|\Theta \right)\prod_{t=1}^{T}P(Y_{t}|\Theta ,X) \end{array}$$ (26)

Where $P(Y_{t}|\Theta ,X)∼N(\beta Z_{t}+H\Phi_{t},\sigma_{\epsilon }^{2}I_{m})$ is the conditional distribution of the infected rate of VLH observation at time t defined by Eq (25). Thus, the posterior marginal distribution of the latent field and the hyper paramater is given by;

$$\begin{array}{c} P(X_{i}|Y)=\int P(X_{i}|\Theta ,Y)P(\Theta |Y)d\Theta \end{array}$$ (27)

$$\begin{array}{c} P(\Theta_{j}|Y)=\int P(\Theta |Y)d\Theta_{-j} \end{array}$$ (28)

for *i* = 1, 2, 3, …, *T* + *p* and *j* = 1, 2, …, 4.

It merits nothing that for the specific model we are managing, described by Gaussian observations, we have that $\tilde{P}(X_{i}|Y)$ is correct and Gaussian and the main approximation is the numerical integration required for computing $\tilde{P}(\Theta |Y)$.

## Results and discussion

### Results

We considered the average weekly infected rate of VLH from the first week of January 2015 to last week of December 2017 with *T* = 156 weeks, at every m = 10 locations and 5 validation sites in Amhara Regional State, Ethiopia, including geographical, socioeconomic, metrological covariates, spatial coordinates and temporal dynamics. We had also average infected rate data and average infected rate validation data with 1560 rows and 780 rows respectively, one row for each week, 15 columns (Station ID, time, the infected rate of VLH, Average number of Household (ANH), Average number of Persons Per housing Unit (ANPPHU), Population Density per square kilometer (PDPSK), Health Facility Coverage in Percentage (HFCIP), Education coverage in percentage (ECIP), Net Migration rate (NMR), Average weekly temperature (AT), Mortality rate (MR), Sex ratio (SR), Clean water coverage in percentage (CWC) and Spatial coordinates (X, Y, Elevation)).

**Fig 5:**
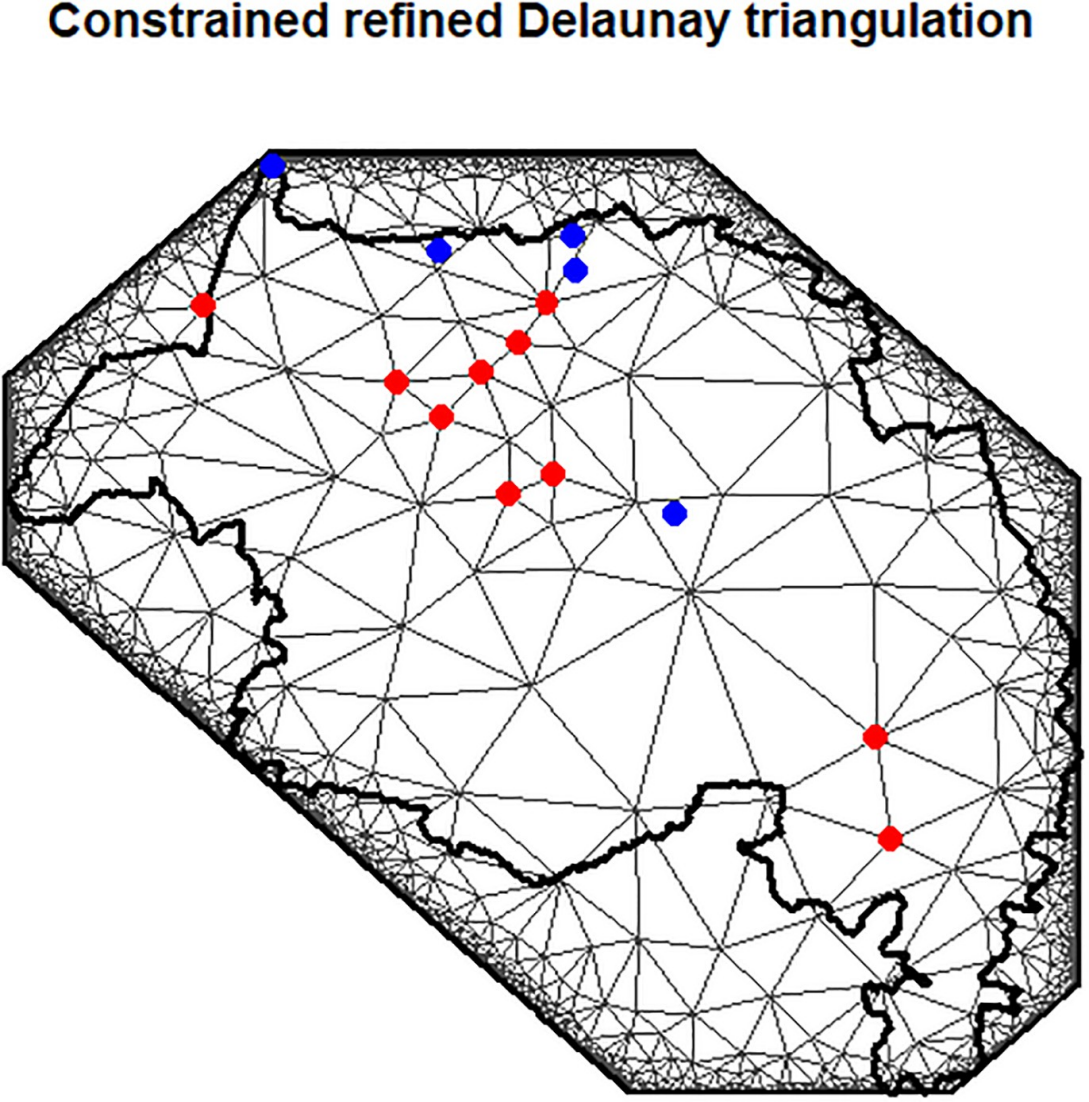
Mesh construction and triangulation of Amhara regional state, for 10 locations and 5 validation sites.

Since the covariates are very extraordinary we applied standardization technique for covariates, Additionally, so as to stabilize the variances, which increment with the mean values, and to make the distribution of the infected rate of VLH data approximately normal, we utilized a logarithmic transformation. The data for this study is provided by the University of Gondar Leishmaniasis research center, Ethiopian Central Statistical Agency (CSA) and Amhara Regional Health Bureau. Fig 5 below demonstrates that the mesh construction and triangulation of Amhara regional state, the red specks showing the area of 10 stations and blue specks shows the validation of 5 stations. We sought a triangulation based on initial vertices at the *m* = 10 station locations and further vertices are included to fulfill the triangulation constraints. We created an SPDE model object for a Matérn-like spatial covariance function with a parameter *α* = 2 implies that the smoothness parameter of Matérn covariance function *v* = 1.

Appreciations to R-INLA, so as to evade having to keep track of vertex indexing, we utilized an R-INLA feature that enables the observation equation to be written in matrix form, *Y* = A*τ* + *ϵ* where, *Y* are the observations, *τ* is a linear predictor, *ϵ* is the observation noise and A is an observation (projection) matrix. The function inla.stack(.) is used to build the necessary data structures, combining simple model building blocks into large complicated models [9] Using a helper function, we built an observation matrix that extracts the values of the spatiotemporal field at the measurement locations and time points used for the parameter estimation. Table 1 above shows that The posterior summary statistics(Mean, Standard Deviation and 95% credible interval) of the fixed effect (covariate coefficient) *β*, Specifically, the posterior mean of the intercept is 0.00247 on the log scale, which corresponds to an average infected rate of visceral leishmaniasis (VLH) is 0.0025, after alteration for covariates.

**Table 1:** Posterior estimates (mean, standard deviation and 95% credible interval) of the covariate coefficient vector *β*.

Covariates	Mean	St.Dev	Quantiles (0.025)	Quantiles (0.5)	Quantiles(0.975)
Intercept	0.00274	3.8869	0.007445	0.002781	0.00293
ANH	0.0019	0.234	-0.00357	0.0019	0.0037
ANPPHU	0.0014	0.176	-0.00127	0.0014	0.0027
PDPSK	-0.0005	0.0700	-0.1379	-0.0005	0.1368
HFCIP	0.0023	0.0051	-0.0099	0.0023	0.0100
ECIP	0.0127	0.0129	-0.0253	0.0127	0.0253
MR	0.0003	0.0401	-0.0785	0.0003	0.0790
AT	0.001	0.0749	0.00015	0.001	0.1469
NMR	0.0012	0.0005	-0.0019	0.0012	0.00329
CWC	0.0005	0.0358	-0.0697	0.0005	0.0707
SR	0.0001	0.0806	-0.1584	-0.0001	0.1580
X	-0.00322	0.034744	-0.0068345	-0.00322	0.0068
Y	-0.00191	0.0291	-0.00572	-0.00191	0.00571
Elevation	-0.00331	0.028319	-0.055689	-0.0041095	0.00556

Expectedly, a significant and positive relationship is perceived between the Average number of Household (ANH) and the Infected Rate of Visceral Leishmaniasis (VLH) since the posterior mean of ANH is 0.0019 and it lies in 95% credible interval. This indicates that an Average number of household (ANH) is one of the determinates of the infected rate of VLH as upheld by the posterior summaries in Table 1 above. Also, a significant and positive relationship is observed between the Average number of Persons Per housing Unit (ANPPHU) and the Infected Rate of Visceral Leishmaniasis (VLH) as the posterior mean of ANPPHU is positive (0.0014) and it lies at 95% credible interval as depicted in posterior summaries above. Additionally, a significant and positive relationship is observed between the Health Facility Coverage in Percentage (HFCIP) and the Infected Rate of Visceral Leishmaniasis (VLH) since the posterior mean of HFCIP is positive (0.0023) and it lies at 95% credible interval, indicates that HFCIP is one of the determinants of the Infected Rate of Visceral Leishmaniasis (VLH). Likewise, there is a significant and positive relationship perceived between Education coverage in percentage (ECIP) and the Infected Rate of Visceral Leishmaniasis (VLH) since the posterior mean of ECIP is positive (0.0127) and it lies at 95% credible interval. As a result, ECIP is also the determinants of the Infected Rate of Visceral Leishmaniasis (VLH) in the region. Similarly, there is a significant and positive relationship observed between Mortality rate (MR) and the Infected Rate of Visceral Leishmaniasis (VLH) since the posterior mean of MR is positive (0.0003) and it lies at 95% credible interval. Correspondingly, there is also a significant and positive relationship observed between Clean water coverage in percentage (CWC) and the Infected Rate of Visceral Leishmaniasis (VLH) since the posterior mean of CWC is positive (0.0005) and it lies at 95% credible interval. As a result, CWC is also another determinant of the Infected Rate of Visceral Leishmaniasis (VLH) in the region. Similarly, there is also a significant and positive relationship observed between Net Migration Rate (NMR) and the Infected Rate of Visceral Leishmaniasis (VLH) since the posterior mean of NMR is positive (0.0012) and it lies at 95% credible interval. Consequently, NMR is also another determinant of the Infected Rate of Visceral Leishmaniasis (VLH) in the region. Additionally, there is also a significant and positive relationship observed between Sex ratio (SR) and the Infected Rate of Visceral Leishmaniasis (VLH) since the posterior mean of SR is positive (0.0001) and it lies at 95% credible interval. Moreover, there is also a significant and positive relationship observed between Average Temperature (AT) and the Infected Rate of Visceral Leishmaniasis (VLH) since the posterior mean of AT is positive (0.001) and it lies at 95% credible interval. Consequently, AT is also another determinant of the Infected Rate of Visceral Leishmaniasis (VLH) in the region and confirms that the importance of meteorological variables on the Infected rate of Visceral Leishmaniasis (VLH). Besides, there is also a significant and negative relationship observed between Population Density per square kilometer (PDPSK) and the Infected Rate of Visceral Leishmaniasis (VLH) since the posterior mean of PDPSK is negative (-0.0005) and it lies at 95% credible interval. Subsequently, PDPSK is also another determinant of the Infected Rate of Visceral Leishmaniasis (VLH) in the region. Finally, there is also a significant and negative relationship observed between, Spatial coordinates (X, Y, Elevation) and the Infected Rate of Visceral Leishmaniasis (VLH) since the posterior mean of (X, Y, Elevation) is negative (-0.00322,-0.00191,-0.00331) respectively and it lies at 95% credible interval. Therefore, Spatial coordinates is also another determinant of the Infected Rate of Visceral Leishmaniasis (VLH) in the region and confirm that the importance of spatial effects on the Infected rate of Visceral Leishmaniasis (VLH).

Table 2 above shows, the summary statistics of the posterior distribution of the AR(1) coefficient λ, the R-INLA function provides us with the mean, quantiles and standard deviation of the Gaussian observation precision parameters $\frac{1}{\sigma_{\epsilon }^{2}}$. As we are apprehensive in the variance $\sigma_{\epsilon }^{2}$, We transformed the marginal density of the precision $\frac{1}{\sigma_{\epsilon }^{2}}$ to variance $\sigma_{\epsilon }^{2}$. The parameter estimates for the spatial SPDE model we obtained using R-INLA which extracts all relevant information from the model summary, also transforms the results from internal parameter scales, giving posterior distributions for nominal variance and nominal range in addition to the internal *θ*_1_ = *log*(*τ*) and *θ*_2_ = *log*(*κ*) where *κ* is a scaling parameter and *τ* is parameter that rescales the field. We get a value of 526 km for the empirically derived correlation range $\rho =\frac{\sqrt{8v}}{\kappa }$. As this is the distance at which the correlation is close to 0.1952, we can conclude that the data are pigeonholed by a strong spatial correlation which diminishes gradually with distance [9]. As depicted by [8], [9] and [23], The Marginal variance, $\sigma_{\gamma }^{2}$ can be approximated with,

$$\begin{array}{c} \sigma_{\gamma }^{2}=\frac{1}{4\pi \kappa^{2}\tau } \end{array}$$

From Table 2 above, $\sigma_{\gamma }^{2}$ was 0.276. We perceived that more variation is explained by the spatial term rather than by the error. Moreover, the high value of the AR(1) temporal correlation coefficient (0.7263) confirms that the short-term persistence of the Infected rate of Visceral leishmaniasis in human(VLH).

**Table 2:** Posterior estimates (mean, standard deviation and 95% credible interval) of the Parametrs $\Theta =(\sigma_{\gamma }^{2},\sigma_{\epsilon }^{2},\lambda ,\rho )$.

Parametrs (Θ)	Mean	St.Dev	Quantiles (0.025)	Quantiles (0.5)	Quantiles(0.975)
$\sigma_{\epsilon }^{2}$	0.0176	0.0081	0.0161	0.0176	0.0193
$\sigma_{\gamma }^{2}$	0.276	0.0035	0.211	0.287	0.321
*ρ*	526	26.1	518	531.6	582
λ	0.7263	0.0273	0.6983	0.7421	0.7916

### Discussion

In this research, we had applied the Stochastics Partial Differential Equation (SPDE) approach for a spatiotemporal hierarchical model for Visceral leishmaniasis in human (VLH) that encompasses a GF and a state process is accompanying with an autoregressive order one temporal dynamics and the spatially correlated error term, together with the effect of land-living cover, metrological, demographic, sociodemographic and geographical covariates in endemic area of Amhara regional state, Ethiopia.

We used a constrained refined Delaunay triangulation (CRDT) for SPDE approximation and mesh construction as a result of the CRDT part is the least relevant as compared to the unconstrained type, it just means that one can specify that certain polygons/line must be part of the triangulation edges. The only constrained edges are usually the boundary edges. The quality of the spde approximation depends mostly on the “refined” aspect. Meshes with small and well-formed triangles (short edges, and no small acute angles) provide closer approximations. Meshes with large and/or sharp angled triangles exhibit approximation artifacts; the fields are always conditionally deterministic, given the values at the mesh vertices. The posterior distributions of covariate effect sizes demonstrate that the mean weekly temperature had a positive association with the infected rate of VLH, This study is in accordance with, the study by [16] in a similar region. Indicates that the outbreak and infection of Visceral leishmaniasis in human (VLH) disease are distributed in the arid and semi-arid parts of the country, high temperatures increase VLH infection rate, this result is in line with the study by [24], [25]. The Average number of Household (ANH) and Average number of Persons Per housing Unit (ANPPHU) had a positive association with the infected the Infected Rate of Visceral Leishmaniasis (VLH). This related to the source of reservoir hosts and human infection anthroponotic leishmaniases, in which the reservoir host is human, the infection and disease transmitted from human to human [1]. Net Migration Rate (NMR) and Mortality rate (MR) had a significant positive association with the Infected Rate of Visceral Leishmaniasis (VLH), this agrees with the study by [6] indicates that migration of laborers and farmers to and from endemic areas increases the infected rate of VLH. Additionally, Health Facility Coverage in Percentage (HFCIP), Education coverage in percentage (ECIP) and Clean water coverage in percentage (CWC) had a significant positive association with the Infected Rate of Visceral Leishmaniasis (VLH), lack of awareness about the disease and symptom, shortage of drug expansions and health facilities and shortage of clean water coverage in the endemic area of Visceral Leishmaniasis (VL) as a result there will be a significant increment of the infection of Visceral Leishmaniasis. Well organized and planned drug expansions in the infected and endemic area, disease controlling mechanisms, better hygiene and sufficient clean water coverage and medical treatments to the infected persons decrease the death and infection rate of VLH. Moreover, Population density per square kilometer (PDPSK) had a significant negative association with the Infected Rate of Visceral Leishmaniasis in humans (VLH), the infection and disease transmitted from human to human, in which the reservoir host is human if the population is very dense and increase the infection of the disease. Sex ratio (SR) also had a significant positive association with on the infected rate of visceral leishmaniasis in human (VLH), Males are more exposed to develop the infection and disease as they are usually engaged in farms, trades and other agricultural activities, which will make them progressively available to the sandfly chomp [6], [26]. From random effects, we get a value of 526 km for the empirically derived correlation range $\rho =\frac{\sqrt{8v}}{\kappa }$. This indicates that the distance at which the correlation is close to 0.1952, we can say that the data are characterized by a strong spatial correlation which diminishes gradually with distance, This result is in line with the study by [20], [27], [28]. $\sigma_{\gamma }^{2}$ was 0.276 and $\sigma_{\epsilon }^{2}$ was 0.0176. We designated that more variation is explained by the spatial term rather than by the error, This result is in line with the study by [20], [29], [30]. Additionally, the high value of the AR(1) temporal correlation coefficient confirms the short-term persistence of Infected rate of Visceral leishmaniasis. There are different possibilities for modifying this study. This study developed dynamic spatiotemporal modeling of the Infected Rate of Visceral Leishmaniasis in humans (VLH) it examined the spatial and temporal effects in addition to the covariate effects through SPDE approach using hierarchical Bayesian modeling. The future studies can modify this study by incorporating the interaction effects of time and space. Also, this study only considered infection of only Infected Rate of Visceral Leishmaniasis in humans (VLH) future studies can extend the study by considering other additional Leishmaniasis infection cases like cutaneous, mucosal or post kala-azar dermal Leishmaniasis (PKDL) in the same study area. We used too many metrological, demographic, sociodemographic and geographic covariates in addition to spatial and temporal effects which lead us model complexity and posterior approximation took too much time. Moreover, future studies can incorporate seasonal dummies to identify the seasonal effects on the infection of Visceral Leishmaniasis in humans (VLH). Lastly, Visceral Leishmaniasis in humans (VLH) case data from stations were very poorly recorded and required very cautious integration. Future studies should modify this study by considering the above limitations in the endemic area of the region.

Even though the above limitations our study identify the spatiotemporal covariates associated with Infected Rate of Visceral Leishmaniasis in humans (VLH) in the region using INLA method. We believe that our modeling result could be used as information and motivation for other studies for identifying spatiotemporal determinants of VLH infection in the endemic areas of Amhara regional state, Ethiopia.

## Conclusions

In this study, we investigated the dynamic spatiotemporal modeling of the infected rate of visceral leishmaniasis (VLH) through SPDE approach. The model involves a Gaussian field (GF) its state process is an autoregressive order one of temporal dynamics and the spatially correlated innovations. Our study had confirmed that both metrological, demographic, sociodemographic and geographic covariates had a significant association with the infected rate of visceral leishmaniasis (VLH) in the region.

Furthermore, Integrated nested Laplace approximations (INLA) is a computationally proficient strategy for incorporating both spatial and temporal effects into spatiotemporal general mixed effect models. Spatiotemporal model fitting is commonly exceptionally complex to execute and requires a powerful computing machine, a long running time, or both. INLA package runs utterly within the commonly utilized R statistical software and is relatively simple to implement with intermediate levels of programming expertise. In ongoing research, we are working on by incorporating the use of expert knowledge of spatiotemporal prediction it just an extension of this work. We also inspire future research in spatiotemporal disease ecology and rare events prediction to consider the INLA SPDE approach for spatiotemporal mixed modeling.

## Supporting information

S1 File S1_File.rar. (RAR)

S2 File R code for posterior estimation. (PDF)
