# Supplementary material for: Dynamic spatiotemporal modeling of the infected rate of visceral leishmaniasis in human in an endemic area of Amhara regional state, Ethiopia
Source: PLoS One. 2019 Mar 1;14(3):e0212934. doi: 10.1371/journal.pone.0212934 (PMC6396920; doi:10.1371/journal.pone.0212934)
Supplement: S2 File — (PDF) [file pone.0212934.s002.pdf]

Anteneh Asmare Godana[1*], Samuel Musili Mwalili[2], George Otieno Orwa[3]

Appendices

R code for posterior estimation for the spatiotemporal modeling fo infected rate of VLH

```
library(INLA)
library(fields)
###memory.limit()
###memory.limit(size=211631800000)
setwd("C:\\Users\\hp\\Desktop\\infected␣rate")
borders <- read.csv("amhara␣region␣border.csv")
head(borders)
index <- dim(borders)[1]
plot(borders)
coordinates<- read.csv("coordinates.csv")
head(coordinates)
coordinates_val<-read.csv("coordinates_Val.csv")
head(coordinates_val)
data<-read.csv("death_weekly.csv")
head(data)
str(data)
data_val<-read.csv("death_val_weekly.csv")
head(data_val)
#str(data_val)
n_stations = length(coordinates$ID) ##10
n_stations_val = length(coordinates_val$ID) ## 5
n_data = length(data$ID ) ## 1560
n_data_val=length(data_val$ID)###980
n_days = as.integer(n_data/n_stations) ## 156
n_days_val=as.integer(n_data_val/n_stations_val)##156
mean_covariates = apply(data[,5:17],2,mean)
sd_covariates = apply(data[,5:17],2,sd)
data[,5:17] = scale(data[,5:17],mean_covariates, sd_covariates)
mean_covariates_val = apply(data_val[,5:17],2,mean)
sd_covariates_val = apply(data_val[,5:17],2,sd)
data_val[,5:17] = scale(data_val[,5:17],
mean_covariates_val, sd_covariates_val)
data$loginfected = log(data$Infected.rate)
data_val$loginfected = log(data_val$infectedrate_val)
data$time = rep(1:n_days, each = n_stations)
data_val$time = rep(1:n_days_val, each = n_stations_val)
mesh = inla.mesh.create.helper
(points=cbind(coordinates$X,coordinates$Y)
,points.domain=borders,offset=c(10, 140),
max.edge=c(50, 1000),min.angle=c(27, 21))
plot(mesh)
lines(borders,lwd=3)
points(coordinates$X,coordinates$Y,pch=20,cex=2, col=2)
points(coordinates_val$X,coordinates_val$Y,pch=20,cex=2,col=4)
spde = inla.spde2.matern(mesh=mesh, alpha=2)
A.est = inla.spde.make.A(mesh,
loc=as.matrix(coordinates[data$ID,c("X","Y")])
```

```
,group=data$time,n.group=n_days)
A.est_val = inla.spde.make.A(mesh,
loc=as.matrix(coordinates[data_val$ID,
c("X","Y")]),group=data_val$time,n.group=n_days_val)
mesh$n
field.indices = inla.spde.make.index("field", n.spde=spde$n.spde,
n.group=n_days)
stack.est=inla.stack
(data=list(y = data$Infected.rate),A=list(A.est, 1),
effects=list(c(field.indices,list(Intercept=1)),
list(ANH=data[,5], ANPPHU=data[,6],PDPSK=data[,7],
HFCIP=data[,8], ECIP=data[,9], MR=data[,10], AT=data[,11],
NMR=data[,12],
CWC=data[,13], SR=data[,14],X=data[,15],
Y=data[,16], Elevation=data[,17])),
tag="est")

stack.val =inla.stack(data=list
(data=list( y = data_val$infectedrate_val),
A=list(A.est_val, 1),
effects=list(c(field.indices,list(Intercept=1)),
list(ANH=data_val[,5], ANPPHU=data_val[,6],
PDPSK=data_val[,7],
HFCIP=data_val[,8],
ECIP=data_val[,9],
MR=data_val[,10],
AT=data_val[,11],
NMR=data_val[,12],
CWC=data_val[,13],
SR=data_val[,14],
X=data_val[,15],
Y=data_val[,16],
Elevation=data_val[,17])),
tag="est_val")

stack = inla.stack(stack.est,stack.val)
formula <- (y~ -1 + Intercept +ANH + ANPPHU  + PDPSK
+ HFCIP +ECIP
+ MR  + AT +
NMR +CWC + SR +X+Y+ Elevation+f(field, model = spde,
group = field.group,
control.group = list(model="ar1")))
result = inla(formula,data = inla.stack.data(stack, spde=spde),
family = "gaussian"
,control.predictor = list(A=inla.stack.A(stack), compute=TRUE))
summary(result)
result$summary.hyperpar["GroupRho␣for␣field",]
sigma2eps_marg = inla.tmarginal(function(x) 1/x,
result$marginals.hyperpar$"Precision␣for␣the␣Gaussian␣observations
sigma2eps_m1 = inla.emarginal(function(x) x, sigma2eps_marg)
sigma2eps_m2 = inla.emarginal(function(x) x^2, sigma2eps_marg)
sigma2eps_stdev = sqrt(sigma2eps_m2 - sigma2eps_m1^2)
sigma2eps_quantiles = inla.qmarginal
(c(0.025, 0.5, 0.975), sigma2eps_marg)
result.field =
inla.spde.result
(result, "field", spde, do.transform=TRUE)
```

```
inla.emarginal(function(x) x, result.field
$marginals.range.nominal[[1]])
inla.emarginal(function(x) x, result.field
$marginals.variance.nominal[[1]])
```
